# Exploring Epigenetic Modifications as Potential Biomarkers and Therapeutic Targets in Glaucoma

**DOI:** 10.3390/ijms25052822

**Published:** 2024-02-29

**Authors:** Emanuele Tonti, Roberto Dell’Omo, Mariaelena Filippelli, Leopoldo Spadea, Carlo Salati, Caterina Gagliano, Mutali Musa, Marco Zeppieri

**Affiliations:** 1 Eye Clinic, Policlinico Umberto I University Hospital, 00142 Rome, Italy; tonti.oculista@gmail.com (E.T.);; 2 Department of Medicine and Health Sciences “Vincenzo Tiberio”, University of Molise, Via Francesco De Sanctis 1, 86100 Campobasso, Italy; 3 Department of Ophthalmology, University Hospital of Udine, 33100 Udine, Italy; 4 Faculty of Medicine and Surgery, University of Enna “Kore”, Piazza dell’Università, 94100 Enna, Italy; 5 Eye Clinic, Catania University, San Marco Hospital, Viale Carlo Azeglio Ciampi, 95121 Catania, Italy; 6 Department of Optometry, University of Benin, Benin City 300238, Nigeria

**Keywords:** glaucoma, epigenetics, neurodegeneration, retinal ganglion cell dysfunction, neurodegenerative disorder, glaucomatous neurodegeneration

## Abstract

Glaucoma, a complex and multifactorial neurodegenerative disorder, is a leading cause of irreversible blindness worldwide. Despite significant advancements in our understanding of its pathogenesis and management, early diagnosis and effective treatment of glaucoma remain major clinical challenges. Epigenetic modifications, encompassing deoxyribonucleic acid (DNA) methylation, histone modifications, and non-coding RNAs, have emerged as critical regulators of gene expression and cellular processes. The aim of this comprehensive review focuses on the emerging field of epigenetics and its role in understanding the complex genetic and molecular mechanisms underlying glaucoma. The review will provide an overview of the pathophysiology of glaucoma, emphasizing the intricacies of intraocular pressure regulation, retinal ganglion cell dysfunction, and optic nerve damage. It explores how epigenetic modifications, such as DNA methylation and histone modifications, can influence gene expression, and how these mechanisms are implicated in glaucomatous neurodegeneration and contribute to glaucoma pathogenesis. The manuscript discusses evidence from both animal models and human studies, providing insights into the epigenetic alterations associated with glaucoma onset and progression. Additionally, it discusses the potential of using epigenetic modifications as diagnostic biomarkers and therapeutic targets for more personalized and targeted glaucoma treatment.

## 1. Introduction

Glaucoma is one of the leading causes of irreversible blindness worldwide; it is a neurodegenerative disease characterized by progressive retinal ganglion cells (RGCs) loss and optic nerve neuroretinal rim degeneration that can lead to severe visual field loss [1]. The global prevalence of glaucoma is 3.54%, with the highest prevalence in Africa. The number of people affected by glaucoma worldwide (aged 40–80 years) will be 111.8 million in 2040 [2]. There are several risk factors for glaucoma onset. Among these are increased intraocular pressure (IOP), family history of glaucoma, genetics, age, gender, race (non-white ethnicity), environmental influences, myopia, pseudoexfoliation, disc hemorrhages, vasospasm, systemic hypotension/hypertension, corticosteroid use and smoking [3,4,5]. Currently, the pathogenesis is not fully clear (i.e., mechanical/ischemic insult, oxidative stress, neuroinflammation, etc.) [6]. 

Despite this multiplicity of risk factors, IOP is the only one on which therapeutic action is possible nowadays. In order to lower IOP, medications, laser therapy, or surgery can be employed. However, several studies highlighted that even glaucomatous patients with IOP within the normal limits will progress in losing RGCs. In addition, novel approaches promoting neuroprotection are now emerging [7,8]. Moreover, among the risk factors, in addition to IOP, those that play a key role in the development of glaucoma are genetics and environmental influences [9]. In that regard, there is an emerging area of scientific research, called epigenetics, that shows how environmental influences affect gene expression. Epigenetics should be recognized as an important element of glaucoma pathogenesis and development [10,11]. Epigenetics, in addition to genetics and environmental factors, influences the signaling pathways that are responsible for disease progression [10,11]. 

In recent years, in fact, there has been an increase in studies on the role of epigenetics in glaucoma that is now considered as an important causal factor in glaucoma. It is known that 16–20% of the risk of primary open-angle glaucoma (POAG) is imputable to genetic factors, and first- and second-degree relatives of affected patients are both at risk [12,13]. The genes involved in the pathogenesis of early-onset glaucoma are OPTN, MYOC, CYP1BI, PAX6, PITX2 and FOXC1; however, mutations in these Mendelian genes account for ≤10% glaucoma cases worldwide. Environmental factors that affect IOP include physical activity, ω-3 and ω-6 fat intake, yoga, and lifting weights [10]. 

Epigenetics can influence the regulation of gene expression with different mechanisms; the three most known and studied ways through which it acts are DNA methylation, histone modification and micro-ribonucleic acids (also called miRNAs). A better understanding of the mechanisms of glaucoma development may provide a therapeutic strategy that is truly effective in blocking or reverting the progression of the disease.

In this review, we analyze how epigenetic modifications can influence gene expression and how these mechanisms are involved in glaucomatous neurodegeneration and contribute to glaucoma pathogenesis. Moreover, the potential for using epigenetic modifications as diagnostic biomarkers and therapeutic targets for more personalized and targeted glaucoma treatment will be discussed. 

This review discusses the role of epigenetic mechanisms in glaucoma, highlighting their significance beyond traditional factors like genetics and intraocular pressure. It emphasizes DNA methylation, histone modifications, and non-coding RNA expression in glaucoma pathophysiology and proposes potential therapeutic targets within the epigenetic realm. Diagnostic biomarkers for early disease detection are also addressed, alongside challenges such as the need for standardized assays and longitudinal validation studies. Overall, the review describes future directions on epigenetic research strategies to enhance glaucoma management and clinical outcomes.

## 2. Mechanisms

### 2.1. DNA Methylation

DNA methylation was detected in mammals as early as DNA was determined as the genetic material [14,15,16]. In 1948, Rollin Hotchkiss first discovered modified cytosine in a preparation of calf thymus using paper chromatography. DNA methylation is an epigenetic mechanism affecting the transfer of a methyl group onto the C5 position of the cytosine to form 5-methylcytosine. DNA methylation regulates gene expression by recruiting proteins involved in gene repression or by inhibiting the binding of transcription factors to DNA [16]. More specifically, DNA methyltransferases (DNMTs) transfer a methyl group from the co-factor S-adenosylmethionine (SAM) to the 5′-position of the cytosine ring in DNA, forming 5mC and completing methylation. 

Demethylation can be passive or active. Passive demethylation occurs due to the lack of functional DNA methylation maintenance mechanisms, while active demethylation is due to the oxidation of 5mC by ten-eleven translocation enzymes (TET) to deliver 5-hydroxymethylcytosine (5hmC), 5-formylcytosine (5fC), and 5-carboxylcytosine (5caC) [17]. DNA methylation levels are intimately related to age; this is an element of debate and study for new therapeutic approaches. Moreover, DNA methylation is necessary for genomic imprinting, X chromosome inactivation, regulating tissue-specific gene expression, and silencing retroviral elements. Additionally, cross-talk between DNA methylation and other epigenetic mechanisms is now known. Interestingly, DNA methylation in distinct genomic regions can influence in different ways gene activities based on the underlying genetic sequence [16,17]. 

Mutations modifying DNA methylation affect metabolic and neurodevelopmental functions and may lead to the evolution of several neurodegenerative diseases [18]. As for glaucoma, the studies showed higher levels of DNA methylation in glaucomatous trabecular meshwork cells. Methylation levels tend to increase in hypoxia states. Moreover, in patients with glaucoma there is an increased expression of TGFβ1 and a reduction in anti-fibrotic factor Rat-sarcoma (RAS) protein activators like RASAL1 [17,19,20]. Elevated levels of TGFβ1concur with the deposition of extracellular matrix in the trabecular meshwork (TM) and the juxtacanalicular region and are also considered a main regulatory factor in the failure of glaucoma filtration surgery [17,21].

Another factor causing the increase in TM fibrosis is given as the demethylation of the GDF7 promoter which induces the transcription of the growth differentiation factor (GDF7) gene, a member of the TGFβ superfamily [22,23,24]. In rhesus macaque models, TM fibrosis was inhibited by antibodies directed against GDF7. In addition, glaucomatous Schlemm’s canal (SC) cells exhibit a remarkably different methylation pattern than control SC cells [25], but more studies are needed to clarify these results. Clearly, all these changes in a pro-fibrotic sense favor the increase in the resistance to the outflow of the aqueous humor and therefore an increase in the IOP. All these new insights serve as a basis for identifying new pharmacological targets.

### 2.2. Histone Modification (Histone Acetylation, Histone Methylation)

A histone is a protein that helps to constitute the structure of chromatin, which is composed of DNA-wrapped protein octamers. Several types of histone modifications are known, amongst which acetylation, methylation, phosphorylation, and ubiquitination are the best studied; such epigenetic modifications have the power to diminish or booster gene expression, mainly as a result of altering chromatin structure [17,26]. Studies on glaucoma-related histone modifications mainly focus on acetylation and methylation [17]. Histone acetylation and histone deacetylation involve the addition or removal of an acetyl group on lysine residues in the N-terminal tail and on the surface of the nucleosome core of histone proteins. 

Acetylated and deacetylated histones are considered epigenetic tags within chromatin by relaxing (euchromatin) or tightening (heterochromatin) chromatin structure, subsequently increasing or decreasing gene transcription levels. Histone methylation causes transcription repression or activation, depending on the target sites. Histone methyltransferases (HMTs) control or regulate DNA methylation through chromatin-dependent transcription repression or activation, depending on the location of the methylation [27]. Measurement of histone methyltransferase activity and quantification of histone methylation patterns have become crucial in studying epigenetic regulation of genes, as well as inhibitor discovery. Histone methylation occurs both on histone lysine and arginine side chains [17]. 

As regards the studies on histone methylation and glaucoma, it was found that trimethylation of lysine 27 on histone H3 (H3K27me3), catalyzed by histone methyltransferase zeste homolog 2 (Ezh2), can mediate epigenetic silencing [28]. H3K27me3 generated under the catalysis of Ezh2 can suppress the PI3K/AKT pathway and downregulate Vgf expression (a neuroprotective factor). Zheng et al. [29] in a pre-clinical study evidenced that inhibiting Ezh2 and H3K27me3 can activate the PI3K-Akt pathway and delay retinal degeneration. Recently, Xiao et al. [30] demonstrated that DZNep (EZH2-specific inhibitor 3-deazaneplanocin A) alters EZH2 and H3K27me3, exerting neuroprotective effects against NMDA-induced retinal neurotoxicity. 

DZNep reduced H3K27me3, upregulated PI3K-Akt and the neuroprotective factor Vgf, reducing NMDA-induced RGC death. The tranylcypromine which is an inhibitor of lysine-specific demethylase 1 (LSD1) was also studied. Tranylcypromine triggers p38 mitogen-activated protein kinase (MAPK) γ which has a proved role as a stress inducer causing pro-inflammatory cytokine production and neurotoxicity [31,32,33]. On the contrary, Tsutsumi et al. in 2016 evidenced that tranylcypromine reduced RGC apoptosis by inhibiting glutamate neurotoxicity and oxidative stress [34]. 

Recent studies on epigenetics and glaucoma suggested that histone acetylation has an important role in increased expression of the glaucoma-associated factor TGFβ2 resulting in damage to TM [35]. Moreover, the inhibition of HDAC (histone deacetylase) supports RGCs survival [17,36]. Several authors claimed that deacetylase inhibitors can preserve and regenerate RGCs in the optic nerve crush model. In these models, glutamate excitotoxicity and the consequent death of the RGC were reproduced [37,38,39,40,41,42]. The limitation of these studies is that they are essentially pre-clinical.

METTL23, a histone arginine methyltransferase expressed in RGCs, which catalyzes dimethylation of histone H3R17 in the retina, when mutated (c.A23G variant) is involved in normal tension glaucoma development [43]. However, additional studies are needed. Indeed, in the study conducted by Pan et al., METTL23 deletion was related to the loss of PS2 and was involved in the NF-κB-mediated activation of TNF-α and IL-1β. So, in the latter study, the methylation on the histone arginine side chain was favorable for RGC survival [43].

### 2.3. Non-Coding RNAs

It is determined that non-coding RNAs (ncRNAs) represent approximately 60% of the genetic material in the human genome [44]. Non-coding RNAs are a cluster of RNAs that do not encode functional proteins and at first were deemed to solely regulate gene expression at the post-transcriptional level [45]. ncRNA epigenetics is mixed up in almost every step of RNA metabolism, regulating stability of ncRNAs, microRNA processing, competing endogenous RNA (ceRNA) network, as well as affecting interaction between long ncRNAs (lncRNAs)and RNA binding proteins [46]. lncRNAs are characterized by a length greater than 200 nucleotides, and their secondary structure allows them to bind to certain proteins to enable chromatin remodeling and modification as well as the linear control of transcription factors. 

Up to now, different lncRNAs have been found to be variously expressed in the aqueous humor, trabecular meshwork, iris and retinal cells, and venous blood of patients with glaucoma [47,48,49]. It has been proved that lncRNA may be a potential biomarker for primary open-angle glaucoma [50,51,52], and studies on lncRNAs mechanism have mostly focused on the ceRNA mechanism. Several studies have pointed out that lncRNA can control retinal ganglion cell loss [53,54,55,56], extracellular matrix deposition, and apoptosis of human trabecular meshwork cells through the ceRNA mechanism [57,58,59,60]. Moreover, a key role in glaucoma epigenetics is linked to microRNAs (also called miRNAs), which can encroach with messenger RNA translation [61]. 

In glaucoma, “protective” microRNAs such as micro-RNA-483-3p reduce extracellular matrix fibrosis in response to stress. On the other hand, disease-promoting microRNAs such as microRNA-100 have been shown to decrease nerve growth, leading to neuroinflammation, oxidative stress, and fibrosis [62,63,64,65]. MiRNAs in aqueous humor that could be possible targets for therapeutic intervention are miR-143-3p, miR-125b-5p, and miR-1260b. The therapeutic potential of miRNAs holds extraordinary promise for the elaboration of novel therapeutic strategies in glaucoma [65]. In preclinical studies, treatment with miRNA mimics (agomirs) or inhibitors (antagomirs) may be a way to increase or lower the expression of selected miRNAs in glaucoma patients and slow the progression of the disease [65,66,67,68].

### 2.4. m6A Methylation

N6-Methyladenosine (m6A) refers to methylation of the adenosine base at the nitrogen-6 position. It is common in prokaryotic genomes and in eukaryotic messenger RNA (mRNA). m6A modifications are dynamic and reversible. There are several factors which are involved in the m6A methylation process, including m6A erasers (FTO and ALKBH5), m6A writers (METTL3, METTL14, METTL16, WTAP, RBM15KIAA1429, and ZFP217), and readers (IGF2BP1,2,3, YTHDF1,2,3, eIF3, YTHDC1,2, HNRNPA2B1, FMR1, and LRPPRC). METTL3 (methyltransferase-like 3) forms a complex with other m6A writers that catalyze m6A methylation. FTO (fat mass and obesity-associated protein) and ALKBH5 (AlkB homolog 5, RNA demethylase) remove the methylase. The reader proteins identify methylated RNA [69]. 

Physiologically, m6A RNA methylation regulates several biological processes, such as gene expression, homeostasis maintenance, cell differentiation/proliferation, lipid and glucose metabolism [70,71,72,73]. On the other hand, m6A modifications are involved in pathological processes (e.g., inflammation, angiogenesis, fibrosis, etc.) [74,75]. m6A RNA dysregulation methylation may be involved in traumatic optic nerve injury (TON) and optic nerve regeneration. 

The m6A RNA methylation may have a key role in the pathogenesis of TON; it is assumed that controlling m6A modification may reduce injury-induced inflammatory responses and cell death in the optic nerve, and that the MAPK and NF-kB signaling pathways can be manipulated to achieve neuroprotective effects [76,77,78,79,80]. Patients with pseudoexfoliative glaucoma (PXG) have a significantly upregulated expression of METTL3 [76]. Moreover, METTL3 could be a potential target to inhibit scar formation after glaucoma filtration surgery, because of its role in human Tenon’s capsule fibroblasts proliferation and extracellular matrix deposition induced by TGF-β1 through Smad3 [81,82]. Similar interactions are shown in Table 1.

## 3. Epigenetic Implications in Glaucoma Neurodegenerative Disease 

Glaucoma is characterized by a progressive loss of retinal germ cells (RGCs) and their axons, which frequently occurs in tandem with elevated intraocular pressure. Evidence from studies conducted on mice, using saline injections as non-glaucomatous controls, unilaterally induced increased intraocular pressure for 21 days by injecting microbeads into the anterior chamber of the eye [83]. Dual adeno-associated viruses (AAVs) were injected intravitreally along with Sox2 and Klf4 genes (OSK) in mouse retinal ganglion cells to restore youthful DNA methylation patterns, and transcriptome expression was induced for an additional 4 weeks, followed by a notable decrease in axonal density and the number of RGCs at the 4-week time point. When compared to glaucomatous eyes that were given AAVs without any OSK induction (−OSK), the glaucomatous eyes that received OSK treatment (+OSK) displayed an axon density that was comparable to the non-glaucomatous eyes [83,84]. 

The concept of “reprogramming” epigenetic information to regenerate tissues or bodily functions represents an innovative frontier, promising to revolutionize the treatment of many ailments, including vision loss. The idea of restoring genetic information to regenerate visual function could pave the way for more effective and less invasive treatments than those currently available. The complex dynamics of epigenetics must be addressed, to understand how epigenetic information is acquired, modified, and transmitted during the cellular reprogramming process. Additionally, ensuring that epigenetic reprogramming is safe and free from risks is necessary, avoiding potential unwanted side effects or complications [85,86,87,88,89].

Another significant challenge concerns the long-term effectiveness of treatments based on epigenetic reprogramming. It is crucial to understand whether the induced epigenetic changes can persist over time and whether vision restoration can be long-lasting or require long-term supportive therapies [90].

Glaucoma epigenetics research has yielded significant insights into the molecular mechanisms underlying the disease, offering novel perspectives on its pathogenesis and potential therapeutic targets. Understanding the results of such research in the context of previous studies and working hypotheses provides a comprehensive view of glaucoma etiology and progression [91].

Firstly, the identification of epigenetic modifications, including changes in DNA methylation, histone modifications, and non-coding RNA expression, has enhanced our understanding of glaucoma pathophysiology. These alterations, observed in various ocular tissues and bodily fluids of glaucoma patients, suggest a widespread dysregulation of gene expression networks, contributing to disease onset and progression [92].

From the perspective of previous studies, the findings of glaucoma epigenetics research underscore the multifactorial nature of the disease. Previous investigations have primarily focused on genetic factors and intraocular pressure as key determinants of glaucoma. However, epigenetic modifications provide additional layers of regulation that intersect with genetic predisposition and environmental factors, shaping the complex landscape of glaucomatous neurodegeneration [93].

Moreover, the implications of these findings extend beyond glaucoma to other neurodegenerative disorders. Epigenetic dysregulation is increasingly recognized as a common feature underlying various neurodegenerative diseases, including Alzheimer’s disease, Parkinson’s disease, and multiple sclerosis. Shared epigenetic signatures across these conditions highlight potential common pathways and therapeutic targets that warrant further exploration [94,95].

In the broadest context, glaucoma epigenetics research highlights the interconnectedness of molecular pathways underlying ocular health and disease. It underscores the importance of considering epigenetic factors in the development of personalized medicine approaches for glaucoma management. By targeting specific epigenetic modifications, it may be possible to modulate gene expression patterns and mitigate neuronal damage, paving the way for more effective therapeutic interventions [95].

The compelling investigation conducted by Pan et al. [46] delineates a genetic etiology underlying normal-tension glaucoma (NTG) and delineates the role of a mutated histone methyltransferase in NTG [96,97,98]. The researchers identified a Japanese family spanning three generations afflicted with NTG and harboring a splicing mutation within the methyltransferase-like 23 (METTL23) gene, responsible for encoding a histone arginine methyltransferase. The autosomal dominant inheritance pattern associated with the METTL23 c.A83G mutation was evident in all six affected family members. This mutation led to aberrant splicing of METTL23 mRNA. Haploinsufficiency of the gene resulted in reduced protein levels and abnormal subcellular localization. Mechanistically, METTL23 catalyzed the methylation of H3R17 in the retina. The estrogen receptor pS2 was identified as a downstream target of this methylation activity, exerting negative regulation on NF-κB signaling [99,100,101,102].

Using a variety of methodologies, including Mettl23-knock-in and -knockout mice, as well as induced pluripotent stem cells (iPSCs) derived from patients with normal-tension glaucoma (NTG), the researchers demonstrated the crucial role of METTL23 in the survival of retinal ganglion cell (RGC) soma and the protection of the optic nerve. Nevertheless, it is plausible that additional factors may influence the phenotype associated with METTL23 heterozygosity [103]. For instance, a METTL23 c.84+60delAT variant, which promotes exon 2 skipping, was more prevalent in NTG patients; however, it was also detected in controls (1.4% of NTG patients and 0.6% of controls), suggesting that METTL23 splice variants may exhibit variable pathogenicity [103,104].

Epigenetics encompasses alterations in gene expression or cellular phenotype without modifying the DNA sequence, influenced by factors like environment, lifestyle, and aging. Various factors may contribute to the association of cataract, which involves lens clouding due to protein aggregation, and glaucoma, characterized by optic nerve damage. Cataracts and glaucoma correlate with oxidative stress, inducing epigenetic changes like DNA methylation and histone modifications, as well as with chronic inflammation, aging, genetic predisposition, and environmental factor links to both conditions, altering epigenetic regulation and gene expression.

## 4. Diagnostic Biomarkers

Identification of diagnostic biomarkers in the epigenetics of glaucoma is paramount for early diagnosis and disease progression monitoring. Recent studies have elucidated the involvement of epigenetic modifications, including DNA methylation, histone modifications, and non-coding RNA expression, in the pathogenesis of glaucoma. For instance, genome-wide DNA methylation profiling has revealed differential methylation patterns between aqueous humor samples obtained from glaucoma patients and healthy controls, suggesting the potential utility of these epigenetic markers in early disease detection [105,106,107,108]. 

Dysregulated expression of histone-modifying enzymes, such as histone deacetylases (HDACs) and histone methyltransferases (HMTs), has been implicated in glaucoma pathogenesis, underscoring their potential as diagnostic biomarkers [109,110,111,112,113,114]. Additionally, aberrant expression of microRNAs (miRNAs) and long noncoding RNAs (lncRNAs) has been observed in glaucomatous tissues and biofluids, highlighting their diagnostic significance [115,116,117,118,119]. Epigenome-wide association studies (EWAS) have identified glaucoma-specific epigenetic signatures, providing insights into disease mechanisms and potential diagnostic targets [120,121,122,123,124] (Table 2).

Moreover, emerging evidence suggests cross-talk between epigenetic modifications and traditional glaucoma risk factors, such as intraocular pressure (IOP) and optic nerve head morphology, further emphasizing the importance of epigenetic biomarkers in glaucoma diagnosis and risk stratification [125,126,127,128,129,130,131,132]. As outlined above, the pathophysiology of glaucoma involves complex interplay between genetic predisposition, environmental factors, and epigenetic alterations. Epigenetic modifications influence gene expression patterns and cellular phenotypes without altering the underlying DNA sequence. In glaucoma, aberrant epigenetic regulation has been implicated in RGC apoptosis, optic nerve degeneration, and neuroinflammation, contributing to disease progression [132]. 

Protein biomarkers linked to glaucoma have been found in a number of areas of the eye’s structure. Care must be taken when interpreting the protein biomarker studies due to their nature, which included the use of various clinical and laboratory methodologies with variable sensitivity and specificity techniques and equipment, too-small sample sizes, numerous unvalidated studies, conflicting reports of dysregulation and a lack of agreement, and a lack of data between the vitreous, aqueous humor, tears, serum, and other samples [133]. Nevertheless, overexpression of the biomarkers may become neurotoxic, and deregulation, as well as a lack or reduced expression of neuroprotectors, will cause the retinal ganglion cells to degenerate through the TrkA receptor pathway. Biomarkers have the potential to offer early screening, diagnosis, and prognostication for glaucoma in the target population. Both the upstream and downstream biomarkers may represent new targets for therapeutic interventions aimed at achieving visual stability or recovery. The blood–aqueous barrier will become distorted as a result of biomarker accumulation because of extracellular matrix tissue dysregulation and inflammation [134,135].

In a similar manner, the biomarkers will interfere with the sympathetic nervous system’s autonomic control, which will impact the physiological architecture of the trabecular meshwork and ciliary body. Over 450 biomarkers have been found to date, but they have never been verified in large sample sizes of patients and controls, nor across various global populations, nor have they been applied in clinical settings, leaving a lot of room for future research [136,137]. Aqueous humor, the optic nerve, the retina, the trabecular meshwork, tears, the vitreous body, serum, and blood have all been found to contain biomarkers. In addition, there exist several biomarkers for immune response, extracellular matrix, oxidative stress, apoptosis, inflammation, neuroprotection, and neurodegeneration [138]. Epigenetic enzymes play a crucial role in regulating gene expression patterns by modifying DNA and histone proteins, thereby influencing chromatin structure and accessibility. Among these enzymes, DNA methyltransferases (DNMTs) and histone-modifying enzymes are particularly significant in orchestrating epigenetic modifications associated with various physiological processes and diseases, including glaucoma [138].

### 4.1. DNA Methyltransferases (DNMTs) 

DNMTs catalyze the addition of methyl groups to cytosine residues within DNA, leading to DNA methylation. This epigenetic modification typically results in gene silencing by altering chromatin structure and inhibiting transcription factor binding. In the context of glaucoma, dysregulation of DNMT activity has been implicated in the aberrant methylation patterns observed in ocular tissues, contributing to disease pathogenesis [139].

### 4.2. Histone-Modifying Enzymes

Histone-modifying enzymes encompass a diverse group of proteins that catalyze post-translational modifications (PTMs) on histone tails, including acetylation, methylation, phosphorylation, and ubiquitination. These modifications modulate chromatin structure and gene expression by altering the accessibility of DNA to transcriptional machinery. In glaucoma, aberrant activity of histone-modifying enzymes, such as histone acetyltransferases (HATs), histone deacetylases (HDACs), histone methyltransferases (HMTs), and histone demethylases (HDMs), has been implicated in neurodegenerative processes and retinal ganglion cell dysfunction [140].

Understanding the intricate interplay between epigenetic enzymes and glaucoma pathophysiology holds promise for identifying novel therapeutic targets and developing personalized treatment strategies for this blinding disease. Integrating epigenetic biomarkers into clinical practice holds promise for improving early detection and personalized management of glaucoma.

## 5. Therapeutic Targets

Exploring therapeutic targets within the realm of epigenetics holds significant promise for the development of novel treatment strategies aimed at preserving vision and halting disease progression. Epigenetic dysregulation, encompassing alterations in DNA methylation, histone modifications, and non-coding RNA expression, plays a pivotal role in the pathogenesis of glaucoma, making it an attractive area for therapeutic intervention.

- DNA Methylation Targets: DNA methyltransferases (DNMTs), the enzymes responsible for DNA methylation, represent promising therapeutic targets in glaucoma. Inhibiting DNMT activity could potentially reverse aberrant DNA methylation patterns associated with glaucomatous neurodegeneration, restoring normal gene expression profiles and mitigating disease severity and impact on the patient’s quality of life [141].
- Histone Modification Targets: Histone-modifying enzymes, including histone acetyltransferases (HATs), histone deacetylases (HDACs), histone methyltransferases (HMTs), and histone demethylases (HDMs), offer additional avenues for therapeutic intervention in glaucoma. Modulating the activity of these enzymes can influence chromatin structure and gene expression, thereby exerting neuroprotective effects and preserving retinal function [142].
- Non-Coding RNA Targets: Non-coding RNAs (ncRNAs), such as microRNAs (miRNAs) and long non-coding RNAs (lncRNAs), have emerged as potential therapeutic targets in glaucoma due to their regulatory roles in gene expression. Targeting specific miRNAs or lncRNAs implicated in glaucoma pathogenesis could offer a targeted approach to modulate disease-associated molecular pathways and mitigate neuronal damage [143].

The cytokine transforming growth factor β (TGF-β) family plays a pivotal role in various cellular mechanisms, including extracellular matrix (ECM) production and accumulation in the trabecular meshwork (TM) and lamina cribrosa (LC). Dysregulation of TGF-β signaling pathways can lead to aberrant ECM remodeling, contributing to increased resistance to aqueous humor outflow and optic nerve damage characteristic of glaucoma. 

A comparative study examining the expression profile of cultured human LC cells revealed that DNA hypomethylation in the promoter region of the TGF-β1 gene led to its enhanced transcription in glaucomatous eye donors compared to controls [101]. This finding underscores the critical involvement of DNA methylation in the onset of glaucoma and in the regulation of TGFβ1 gene expression, offering novel therapeutic avenues [144]. The regulation of fibrosis and hypoxia processes holds significant importance in the management of glaucoma. In a rabbit model of glaucoma filtration surgery (GFS), the purpose of the study was to evaluate the effectiveness of suberoylanilide hydroxamic acid (SAHA), a histone deacetylase inhibitor (HDACi), in preventing excessive wound healing and scar formation [145].

The standard procedure for the clinical management of drug-refractory glaucoma is still glaucoma filtration surgery (GFS). Despite advances in minimally invasive techniques and alternative treatments, such as laser trabeculoplasty and minimally invasive glaucoma surgery (MIGS), glaucoma filtration surgery remains a mainstay due to its efficacy in lowering intraocular pressure and halting disease progression. However, its invasive nature and potential for complications necessitate careful patient selection and ongoing monitoring to optimize outcomes and minimize risks associated with surgical intervention [145]. The two main obstacles to the surgical success of GFS are scarring of the conjunctiva overlying the wound and postoperative wound healing of the scleral flap. SAHA significantly lowers postoperative scarring in the GFS rabbit model. The groups that received SAHA treatment exhibited consistently larger bleb areas, which was further supported by histologic findings indicating a reduction in collagen and ECM deposits. Histone acetylation may be involved in transcriptional regulation, most likely through modifications to chromatin structures, according to multiple lines of evidence. The more highly acetylated isoforms of core histones are also enriched in chromatin fractions that are enriched in actively transcribed genes. Direct binding of suberoylanilide hydroxamic acid occurs at the catalytic site [146].

Targeting enzymes involved in chromatin remodeling, such as SWI/SNF complexes and ATP-dependent chromatin remodelers, holds promise for modulating the epigenetic landscape in glaucoma [147]. Moreover, emerging evidence suggests that targeting epigenetic regulators may synergize with existing therapeutic approaches, such as intraocular pressure-lowering medications and neuroprotective agents, to achieve optimal treatment outcomes [148]. Recent studies have focused on identifying specific epigenetic biomarkers associated with glaucoma onset and progression [149]. For instance, Sharma et al. demonstrated aberrant DNA methylation patterns in ocular tissues of glaucoma patients, suggesting their potential utility as diagnostic and prognostic biomarkers. Furthermore, analysis of histone modifications and non-coding RNA profiles has revealed additional candidate biomarkers with diagnostic and therapeutic implications [150]. The integration of therapeutic biomarkers into clinical practice holds significant promise for personalized management of glaucoma.

By stratifying patients based on their epigenetic profiles, clinicians can tailor treatment strategies to target specific molecular pathways implicated in glaucomatous neurodegeneration. However, several challenges remain, including the need for standardized biomarker assays, longitudinal validation studies, and ethical considerations regarding patient privacy and data sharing [150]. Future research efforts should focus on elucidating the functional significance of identified biomarkers and their potential as therapeutic targets. Integrating multi-omics approaches, such as epigenome-wide association studies (EWAS) and single-cell sequencing, can provide comprehensive insights into the dynamic epigenetic landscape of glaucoma progression. Additionally, collaborative initiatives aimed at data sharing and establishing large-scale biobanks are essential for accelerating biomarker discovery and translation into clinical practice therapeutic biomarkers in glaucoma epigenetics and represent a promising avenue for advancing precision medicine approaches in the management of this sight-threatening disease.

By harnessing the power of epigenetic profiling, clinicians can achieve earlier diagnosis, monitor disease progression more accurately, and develop personalized therapeutic interventions to preserve vision and improve patient outcome [151]. Furthermore, exploring the intricate interplay between genetic predisposition, environmental factors, and epigenetic modifications could unveil novel mechanisms underlying glaucoma pathogenesis. Longitudinal studies tracking epigenetic changes over time in glaucoma patients may provide valuable insights into disease progression and response to treatment. Moreover, leveraging artificial intelligence algorithms for data analysis and pattern recognition in large-scale epigenomic datasets holds tremendous potential for identifying predictive biomarkers and optimizing therapeutic strategies.

The integration of cutting-edge technologies and interdisciplinary collaborations will be paramount in advancing our understanding of glaucoma epigenetics and translating these findings into clinical practice [151]. Ultimately, a personalized medicine approach tailored to the individual epigenetic profile of glaucoma patients promises to revolutionize disease management, offering improved outcomes and enhanced quality of life. As we delve deeper into the complexities of glaucoma epigenetics, continued research and innovation will be crucial for unlocking its full therapeutic potential and addressing the unmet needs of patients worldwide.

By elucidating the intricate mechanisms underlying epigenetic regulation in glaucoma, it is possible to identify druggable targets and develop precision therapies tailored to individual patients. However, translating these discoveries from bench to bedside requires rigorous preclinical validation and clinical trials to ensure safety, efficacy, and long-term therapeutic benefits. The pursuit of epigenetic-based therapies for glaucoma represents a paradigm shift in ocular disease management, offering new hope for patients afflicted by this sight-threatening condition. Table 3 shows a summary table of glaucoma epigenetic insights.

## 6. Conclusions and Future Directions

Unlike genetic mutations, epigenetic alterations possess reversible attributes. This reversibility of epigenetic modifications introduces a novel prospect for investigating potentially more efficacious treatment modalities for glaucoma. In the past few years, there has been notable progress in understanding the epigenetic mechanisms underlying glaucoma that is frequently diagnosed at advanced stages, often after irreversible vision impairment has set in. Epigenetic alterations, such as changes in non-coding RNAs, m6a methylation, and DNA methylation, have been identified with notable shifts in expression levels in various bodily fluids like aqueous humor, tears, peripheral blood, and human trabecular fibroblasts (HTFs) among glaucoma patients and those undergoing glaucoma filtration surgery. These findings unveil numerous potential targets for clinical interventions aimed at preventing, treating, and diagnosing glaucoma at early stages.

It is crucial to recognize that glaucoma is a complex, multifactorial condition, and regulating a single factor may not entirely elucidate the damage inflicted by glaucoma. Furthermore, studies have verified that one epigenetic mechanism can influence another, highlighting the intricate interplay within glaucoma pathogenesis [151]. Future research directions in glaucoma epigenetics should focus on elucidating the causal relationships between epigenetic alterations and disease progression. Longitudinal studies examining epigenetic changes over time and in response to therapeutic interventions are needed to establish causality and identify predictive biomarkers of disease onset and progression. Additionally, exploring the crosstalk between different epigenetic mechanisms and their interaction with environmental factors will provide deeper insights into glaucoma pathogenesis. In summary, glaucoma epigenetics research represents a paradigm shift in our understanding of the disease, offering new avenues for early diagnosis, targeted therapy, and personalized medicine. By integrating findings from diverse disciplines and embracing emerging technologies, we can unlock the full potential of epigenetics in revolutionizing glaucoma management and improving patient outcomes.

## Figures and Tables

**Table 1:** Glaucoma epigenetic mechanisms.

Glaucoma Epigenetic Modifications	Mechanisms	Main Link to Glaucoma
DNA methylation	DNA methylation regulates gene expression, recruiting proteins involved in gene repression or by inhibiting the binding of transcription factors to DNA	Higher levels of DNA methylation have been found in glaucomatous trabecular meshwork cells. Methylation levels tend to increase in hypoxia states and enhance fibrotic factors expression [15]
Histone modification	Histone methylation and histone demethylation are epigenetic modifications that have can reduce or enhance gene expression, especially as a result of altering chromatin structure [17]	Controversial role addressed in the main text → additional studies are needed
Non-coding RNAs	Non-coding RNAs can act as modulators of epigenetics through chromatin remodeling. NcRNAs can regulate gene expression at transcriptional or post-transcriptional level	lncRNA may be a potential biomarker for primary open-angle glaucoma lncRNA can control retinal ganglion cell loss, extracellular matrix deposition, apoptosis of human trabecular meshwork cells through the ceRNA mechanism. “Protective” microRNAs such as micro-RNA-483-3p reduce extracellular matrix fibrosis in response to stress disease-promoting microRNAs such as microRNA-100 and have been shown to decrease nerve growth leading to neuroinflammation, oxidative stress, and fibrosis [61]
m6A methylation	m6A methylation can regulate the structure, stability, splicing, export, transcription and deterioration of mRNA, miRNA and lncRNA through methyltransferases, demethylases and m6A binding proteins	Controlling m6A modification may reduce injury-induced inflammatory responses and cell death in the optic nerve METTL3 (methyltransferase-like 3) is upregulated in patients with pseudoexfoliative glaucoma [73]

**Table 2:** Glaucoma-specific epigenetic biomarkers.

Biomarker	Diagnostic Role
DNA Methylation	Elevated DNA methylation levels Specific CpG island hypermethylation Altered methylation patterns in ocular tissues and bodily fluids [97]
Histone Modifications	Aberrant histone acetylation levels Dysregulated histone methylation Altered histone phosphorylation [114]
Non-coding RNAs	Dysregulated expression of microRNAs Abnormal levels of long non-coding RNAs ^1^ Dysregulated expression of circRNAs [118]

^1^ Diagnostic biomarkers associated with glaucoma epigenetics.

**Table 3:** Glaucoma Epigenetics Insights, Diagnostic Biomarkers, and Therapeutic Targets.

	Summary
Glaucoma Epigenetics Insights	- Glaucoma involves progressive loss of retinal ganglion cells (RGCs) and axons, often associated with elevated intraocular pressure. - Epigenetic reprogramming shows promise in protecting against axonal loss and RGC degeneration. - Epigenetic dysregulation is common in various neurodegenerative diseases.
Diagnostic Biomarkers	- Elevated DNA methylation levels, specific CpG island hypermethylation, and altered methylation patterns are observed in ocular tissues and bodily fluids of glaucoma patients. - Aberrant histone modifications and dysregulated non-coding RNA expression are also potential diagnostic markers.
Therapeutic Targets	- Inhibiting DNMT activity can reverse aberrant DNA methylation patterns associated with glaucomatous neurodegeneration. - Modulating histone modification enzyme activity can preserve retinal function and exert neuroprotective effects. - Targeting specific non-coding RNAs offers a tailored approach to mitigate neuronal damage and modulate disease-associated pathways.

## Data Availability

Not applicable.

## Abbreviations

**Abbreviation**	**Definition**
RGC(s)	Retinal Ganglion Cell(s)—Nerve cells located in the retina that transmit visual impulses to the brain.
FTO	Fat Mass and Obesity Associated—Gene involved in obesity and lipid metabolism.
ALKBH5	AlkB Homolog 5—Protein involved in RNA demethylation.
METTL3	Methyltransferase Like 3—Enzyme involved in RNA methylation.
WTAP	Wilms Tumor 1 Associated Protein—Protein involved in regulation of RNA maturation.
RBM15KIAA1429	RNA Binding Motif Protein 15 and KIAA1429—Proteins involved in RNA methylation regulation.
ZFP217	Zinc Finger Protein 217—Protein involved in gene expression regulation.
IGF2BP1,2,3	Insulin-like Growth Factor 2 mRNA Binding Protein 1,2,3—Proteins involved in mRNA stabilization.
YTHDF1,2,3	YTH Domain Containing Family Protein 1,2,3—Proteins involved in mRNA regulation.
eIF3	Eukaryotic Initiation Factor 3—Protein complex involved in initiation of mRNA translation.
YTHDC1,2	YTH Domain Containing 1,2—Proteins involved in RNA regulation.
HNRNPA2B1	Heterogeneous Nuclear Ribonucleoprotein A2B1—Protein involved in RNA splicing.
FMR1	Fragile X Mental Retardation 1—Gene associated with Fragile X mental retardation syndrome.
LRPPRC	Leucine Rich Pentatricopeptide Repeat Containing—Protein involved in RNA regulation and mitochondrial metabolism.
